# Growth and wear characteristics of individual claws in young dairy calves

**DOI:** 10.3168/jdsc.2023-0408

**Published:** 2024-01-15

**Authors:** A.F. Souza, R.L. Wallace, D.J. Tomlinson, T. Earleywine, M.T. Socha, J.K. Drackley, J.S. Osorio

**Affiliations:** 1 School of Animal Sciences, Virginia Tech, Blacksburg, VA 24061; 2 Department of Veterinary Clinical Medicine, University of Illinois, Urbana, IL 61801; 3 Zinpro Performance Minerals, Eden Prairie, MN 55344; 4 Land O'Lakes Animal Milk Products Inc., Shoreview, MN 55126; 5 Department of Animal Science, University of Illinois, Urbana, IL 61801

## Abstract

Data are limited on how cow hooves develop early in life and how this will affect susceptibility to lameness and claw disorders later in life. The aim of this study was to characterize individual claw growth and wear in dairy calves. A total of 90 male Holstein calves <1 wk old were enrolled in this completely randomized design experiment and monitored until wk 20 of age. Evaluation of hoof development was conducted by visual inspection, and individual claw measurements were taken with a graduated ruler. Claws were numbered from 1 to 8 to maintain consistent data collection, where front claws were numbered 1 to 4 (left to right) and rear claws were 5 to 8. Lateral claws were numbered 1, 4, 5, and 8, and medial claws were 2, 3, 6, and 7. Measurements included length from the coronary band to the end of the hoof wall and from the coronary band to the groove line, resulting in the variables claw length (CL) and groove length (GL), respectively. Hoof development was evaluated at wk 0, 5, 10, 15, and 20. All evaluations were performed by the same person throughout the study to eliminate variation between observers. Hoof growth and wear were derived from CL and GL measurements and analyzed from wk 0 to 20. Claw length at wk 0 was different across claw positions, and maximal claw length was observed in claws in positions 6 and 7 (i.e., rear medial claws). A similar growth pattern in claw length was observed when evaluated from wk 0 to 20, where a greater claw length was observed in claws 6 and 7 compared with other claws. In contrast to claw length, claw wear at wk 20 was lower in claws 6 and 7 compared with other claws. Compared with rear claws, front claws had greater wear at wk 20, and compared with medial claws, lateral claws had greater wear at wk 20. Factors associated with claw length differences at wk 0 remain to be elucidated, but the fact that this uneven claw length difference was maintained 20 wk after birth is puzzling. The complementary claw length to wear described greater wear in claws 1 and 4 (i.e., front lateral) in young calves, which is translated into lower claw length in the same claws by wk 20 after birth. Future research in postnatal hoof growth should strive to understand the potential biological significance of this effect on lameness resistance and longevity of dairy cows.

## Highlights

• Claw length at week 0 differed across claw positions, with the maximal claw length observed in claws in positions 6 and 7.
• Claw length evaluated until 20 weeks of age revealed that a greater claw length was maintained in claws 6 and 7 compared with other claws.
• Claw wear at 20 weeks of age was lower in claws 6 and 7 compared with other claws.
• At 20 weeks of age, front claws had greater wear than rear claws, and lateral claws had greater wear than medial claws.
• Factors associated with claw length differences at week 0 remain to be elucidated, but it is puzzling that this uneven claw length difference was maintained 20 weeks after birth.

## Graphical Abstract

**Figure:**
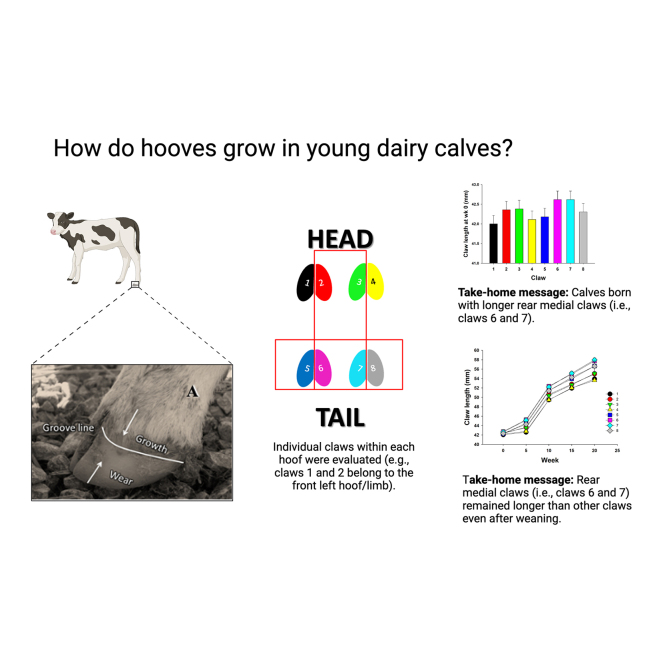
**Summary:** The objective of this study was to characterize individual claw growth and wear in dairy calves. We tracked hoof development in 90 male Holstein calves from 1 to 20 weeks of age. Hoof development was evaluated through visual inspection, and individual claw measurements were recorded using a graduated ruler. At week 0, we observed differences in claw length across different positions, with the rear medial claws (claws 6 and 7) being the longest. This elongation of the rear medial claws persisted until 20 weeks of age. Interestingly, claw wear at week 20 was lower in the rear medial claws compared with other claws. Front claws experienced more wear than rear claws, and lateral claws exhibited greater wear than medial claws. Furthermore, claws 1 and 4 (front lateral) showed increased wear, leading to reduced claw length by week 20. The factors contributing to the observed claw length differences at week 0 remain to be determined.

Lameness is a major cause of economic losses in the dairy industry (Hendry et al., 1997; Green et al., 2002; Donovan et al., 2004). This condition is associated with decreased milk production and fertility, along with increased treatment costs and involuntary culling (Neveux et al., 2006). Changes in hoof conformation in dairy cows during lactation have been associated with lameness and claw lesions (Offer et al., 2000); however, there are limited data on how hoof development early in life affects hoof conformation and lameness susceptibility later in life.

Replacement of heifers in the dairy industry, especially in the United States, is a topic of interest because of the economic impact a heifer raising program can have on any dairy farm income. This has implications for mortality rate, delayed pregnancy, and decreased first lactation milk yield. Previous research in dairy calves has evaluated the effects of the plane of nutrition on overall performance preweaning (Diaz et al., 2001; Hammon et al., 2020), postweaning (Brown et al., 2005), and during first lactation (Terré et al., 2009; Soberon et al., 2012; Gelsinger et al., 2016). The latter aimed to find the optimal growth rate to prepare the future cow for maximal production and resilience to withstand stress periods. However, it remains to be elucidated how early nutrition and management programs in dairy calves could affect hoof health and lameness problems during future lactations. Because of these gaps in knowledge, our objective was to characterize individual claw growth and wear in dairy calves. Our hypothesis was that individual claws will have different growth and wear rates as the calf grows, which can be associated with increased BW and weight distribution.

The University of Illinois Institutional Animal Care and Use Committee approved all procedures for this study. Experimental design and management procedures have been previously described by Osorio et al. (2012). Briefly, 90 male Holstein calves less than 1 wk old were purchased by a buyer in 3 groups of 30 to 35 calves from farms in southern Wisconsin and transported via livestock trailer to the Nutrition Field Laboratory site at the University of Illinois. Calves were housed in individual hutches from arrival through wk 9 and then group housed in super hutches from wk 10 to 12. At wk 13, calves were transported (<0.5 h) to the University of Illinois Beef Research Unit for housing through wk 20. From arrival through wk 12, straw was used as bedding over a base of crushed limestone; then, at the Beef Research Unit, calves were housed in pens on rubber-covered concrete slatted floors.

Dietary treatments have been explained previously (Osorio et al., 2012). Briefly, treatments were combinations of either low or high plane of nutrition, with either inorganic or organic sources of supplemental trace minerals. Compared with low plane of nutrition, the high plane of nutrition treatment consisted of greater amounts and nutrient density of milk replacer and starter, as well as less forage during the growing phase. For the organic trace mineral treatment, organic forms of Zn, Mn, Cu, and Fe were supplemented in milk replacers at 50, 50, 10, and 100 mg/kg, respectively, whereas the inorganic treatment contained the sulfate forms of these trace minerals at the same concentrations. In starter and grower concentrates, organic or inorganic forms of Zn, Mn, Cu, and Co were provided at 70, 55, 12, and 1 mg/kg, respectively. Calves received milk replacer at varying amounts and starter grain ad libitum to induce dietary effects evaluated by Osorio et al. (2012). However, for the purpose of the current study, dietary effects were disregarded. Milk replacers (manufactured by Land O'Lakes Animal Milk Products Inc., Shoreview, MN) contained only milk-derived proteins and were not medicated. Weaning procedures were according to the standard recommendations of the milk replacer manufacturer and consisted of decreasing milk replacer feeding to once a day during the week preceding weaning. Calves remained in their individual hutches from wk 0 through wk 9. Milk replacer, starter mix, and grower grain were sampled throughout the study.

Evaluation of hoof development was carried out by visual inspection and individual claw measurements were taken with a graduated ruler with minimum units in millimeters. Claw measurements, including claw length (**CL**) and groove length (**GL**) were taken to determine the growth and wear of claws (Figure 1A; Vermunt and Greenough, 1995). Claw length was measured from the coronary band to the end of the hoof wall, where the hairline between the coronary band and the horny hoof wall was used as the reference point for the beginning of the coronary band. The groove line was measured from the coronary band to the groove line (Figure 1A). Claw measurements were taken directly down the anterior (cranial) surface of the claw (from hairline to tip of toe) so that it was consistent across calves and time points. Claws were numbered from 1 to 8 to maintain consistent data collection, where front claws were numbered 1 to 4 (from left to right) and rear claws were 5 to 8. Lateral claws were enumerated 1, 4, 5, and 8, and medial claws were 2, 3, 6, and 7 (Figure 1B). Hoof development was evaluated at wk 0, 5, 10, 15, and 20. All evaluations were performed by the same trained veterinarian throughout the study to eliminate variation between observers. Groove length was not measurable until wk 10 due to the poorly defined groove line before that time. Additional trait measures were evaluated, including hoof angle, heel angle, and heel depths; however, variation within claw at the same time point was too high to make these measurements useful throughout the study. Hoof growth and wear were analyzed from wk 0 to 20 based on an adaptation of the procedures described in Hahn et al. (1986). Total hoof growth was assumed to be GL at wk 20, and total wear was calculated by subtracting the remnant claw length (Figure 1A) below the groove line at wk 20 from the initial claw length at wk 0. Data were analyzed as either repeated measures from 0 to 20 wk or as single time points at 0 and 20 wk. Data were analyzed using the MIXED procedure of SAS (version 9.2; SAS Institute Inc., Cary, NC) as repeated measures. The mixed effects model was

*Y_i_* = *µ* + *B_i_* + *P_j_* + *T_k_* + *PT_jk_* + *C*_(_*_i_*_)_*_l_* + *e_ijklm_*,

where *Y_i_* is the dependent continuous variable; *µ* is the overall mean; *B_i_* is the random effect of group (*i* = 1, 2, 3); *P_j_* is the fixed effect of claw (*j* = 1, 2, . . . 8); *T_k_* is the fixed effect of week (*k* = 0, 5, 10, 15, 20); *PT_jk_* is the fixed effect of the interaction between the *j*th claw effect and the *k*th week; *C*_(_*_i_*_)_*_l_* is the random effect of *l*th calf nested with the *i*th group; and *e_ijklm_* is the random residual ~*N* (0, *σ*^2^). The first-order autoregressive covariance structure was selected from among others (e.g., compound symmetry and heterogeneous autoregressive 1) due to its smaller Bayesian information criteria values. Claw length at wk 0 was used as a covariate in the model. Data collected at wk 20, including total growth and total wear, were analyzed using the same model previously described without the repeated statement for the *T_k_* fixed effect of week. Initial claw length was maximal in claw positions 6 and 7 (i.e., rear medial claws; Figure 1C). Therefore, a contrast statement was used to assess this effect on hoof growth and wear over time. Additional contrast statements were used to evaluate differences between front versus rear and medial versus lateral claws. Statistical significance and tendencies were declared at *P* ≤ 0.05 and 0.05 ≤ *P* ≤ 0.10, respectively.

**Figure 1:**
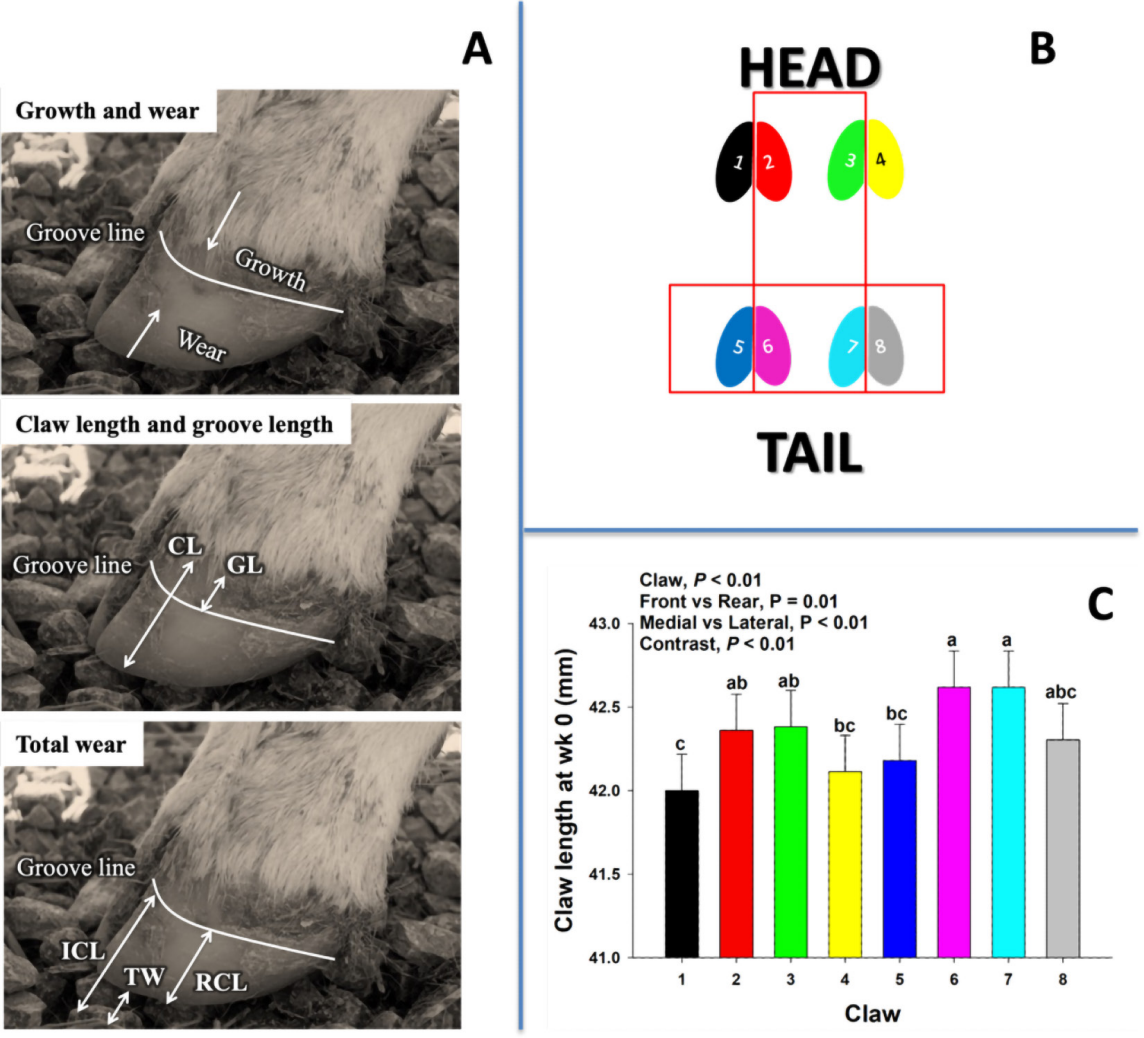
(A, top) General depiction of hoof growth and wear relative to the groove line and coronary band. The hairline was used as the reference point for the beginning of the coronary band. (Middle) Measurements of claw length (CL) and groove length (GL) over the hoof wall. (Bottom) Total wear (TW) in wk 0 to 20 was calculated by subtracting the remnantclaw length (RCL) below the groove line at wk 20 from the initial claw length (ICL) at wk 0. (B) Claws were numbered 1 to 8 to maintain consistent data collection, with front claws numbered 1 to 4 (left to right) and rear claws 5 to 8. Lateral claws were numbered 1, 4, 5, and 8, and medial claws were 2, 3, 6, and 7. (C) Claw length at wk 0 for each claw position with contrast statements to compare front versus rear and medial versus lateral claws. The contrast *P*-value denotes the comparison between claw 6 and 7 against all other claws. (a–c) Means without a common lowercase letter are different (*P* < 0.05). Values are means, with SE represented by error bars.

On average, claw length for each claw at wk 0 had a standard deviation and coefficient of variation of 2.03 and 4.8, respectively. Furthermore, the standard deviation ranged from 1.9 to 2.2 for all claws, while the CV ranged from 4.4 to 5.3. This suggests a low dispersion and variability of claw length at wk 0.

Claw length at wk 0 was different (*P* < 0.01) across claw positions, and maximal claw length was observed in claw positions 6 and 7 (Figure 1C). A contrast statement was used to perform a comparison between claws 6 and 7 against all the other claws, which confirmed the greater (*P* < 0.01) length in claws 6 and 7 in neonatal dairy calves. A similar pattern in claw length was observed when evaluated throughout the study from 0 to 20 wk, where a greater (*P* < 0.01) claw length over time was observed in claws 6 and 7 than in all other claws (Figure 2A). The latter was reflected at 20 wk, where claw length in claws 6 and 7 was greater (*P* < 0.01) than in all other claws (Figure 2B).

**Figure 2:**
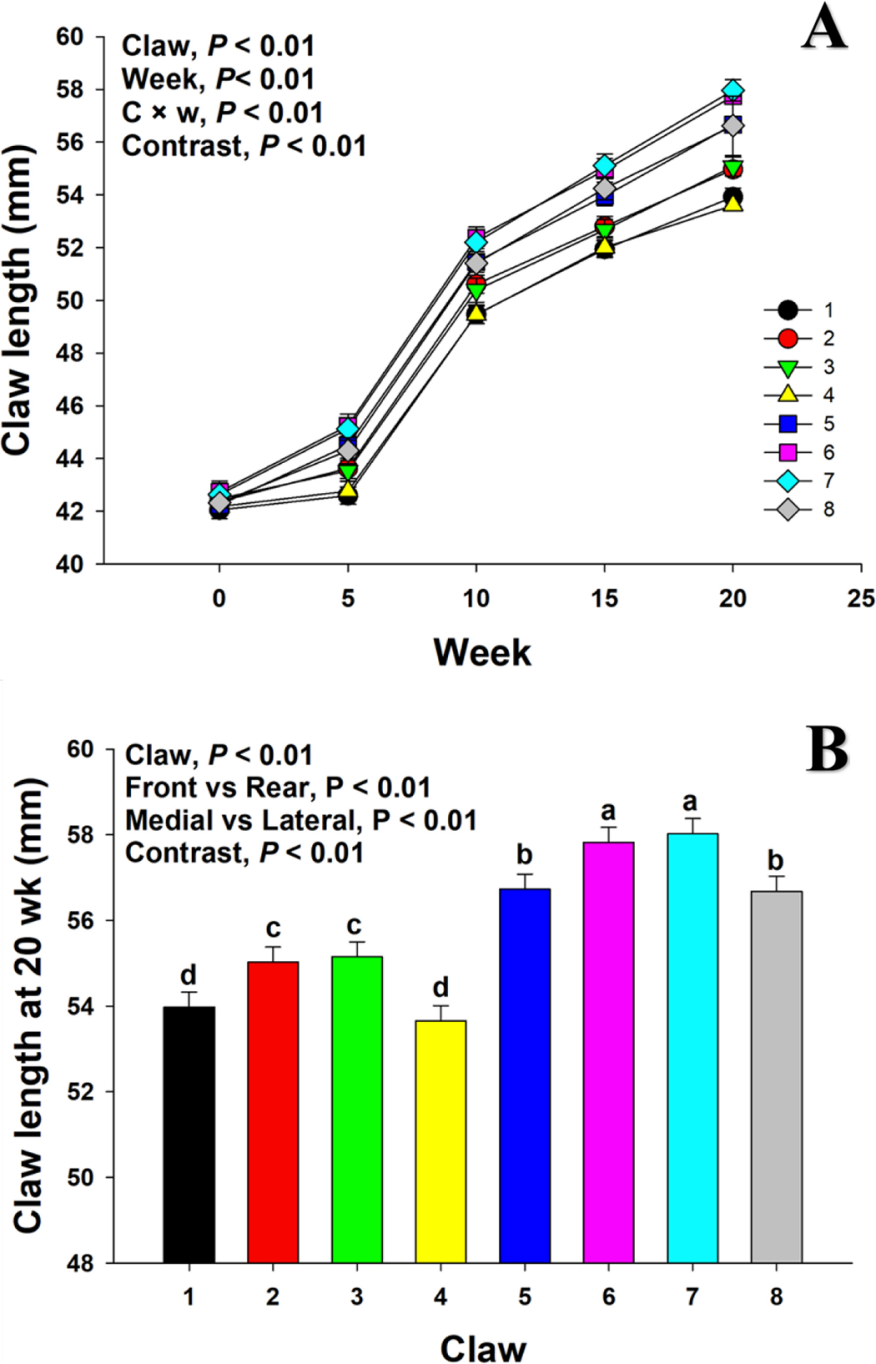
(A) Covariate adjusted means for claw length for each claw, wk 0 to 20. C × w = claw by week interaction. (B) Claw length at 20 wk for each claw position, with contrast statements to compare front versus rear and medial versus lateral claws. The contrast *P*-value denotes the comparison between claws 6 and 7 against all other claws. (a–d) Means without a common lowercase letter are different (*P* < 0.05). Values are means, with SE represented by error bars.

The asymmetry between lateral and medial claws in neonatal calves has been previously reported by radiography in distal limb bone length (Muggli et al., 2011). In the current study, a greater (*P* < 0.01) medial claw length was consistently observed in comparison to lateral claws at wk 0 (Figure 1C; 42.5 vs. 42.1 mm) and 20 (Figure 2B; 56.5 vs. 55.2 mm). In contrast, Muggli et al. (2011) observed that lateral claws were longer than medial claws. This discrepancy may be related to the type of parameter used, bone length in Muggli et al. (2011), and claw length in the current study. However, in adult animals, it is commonly observed that rear lateral claws grow at a faster rate than rear medial claws (Shearer and van Amstel, 2001). Furthermore, Raven (1989) argued that, due to a wide variation in weight bearing, the lateral rear claws grow faster than the medial rear claws, which have a more constant weight bearing and, consequently, greater wear. This effect was previously confirmed by Hahn et al. (1984), where a higher growth in lateral rear claws compared with medial rear claws was observed in breeding heifers and adult cows during a 20-mo period. In the present study, 20-wk-old calves had longer medial claws than lateral claws, regardless of the front or rear position (Figure 2B). This is contrary to the higher growth of lateral rear claws suggested by Raven (1989) and Hahn et al. (1984). Taken together, these effects could be ascribed to a possible change in weight distribution that occurs as the calf grows. Early in life, calves have longer medial claws, and over time a change in weight distribution might promote longer lateral rear claws in mature animals.

Total growth was considered as the groove length at wk 20 for each claw (Figure 3A). Interestingly, although a claw effect (*P* = 0.01) was observed for total growth, this effect was perhaps influenced by the difference in total growth between claws 4 and 5 (Figure 3A; 29.5 vs. 30.3 mm, respectively). Similarly, the trend (*P* = 0.06) for greater total growth in rear claws compared with front (30.1 vs. 29.9) could be partially attributed to the difference between claws 4 and 5.

**Figure 3:**
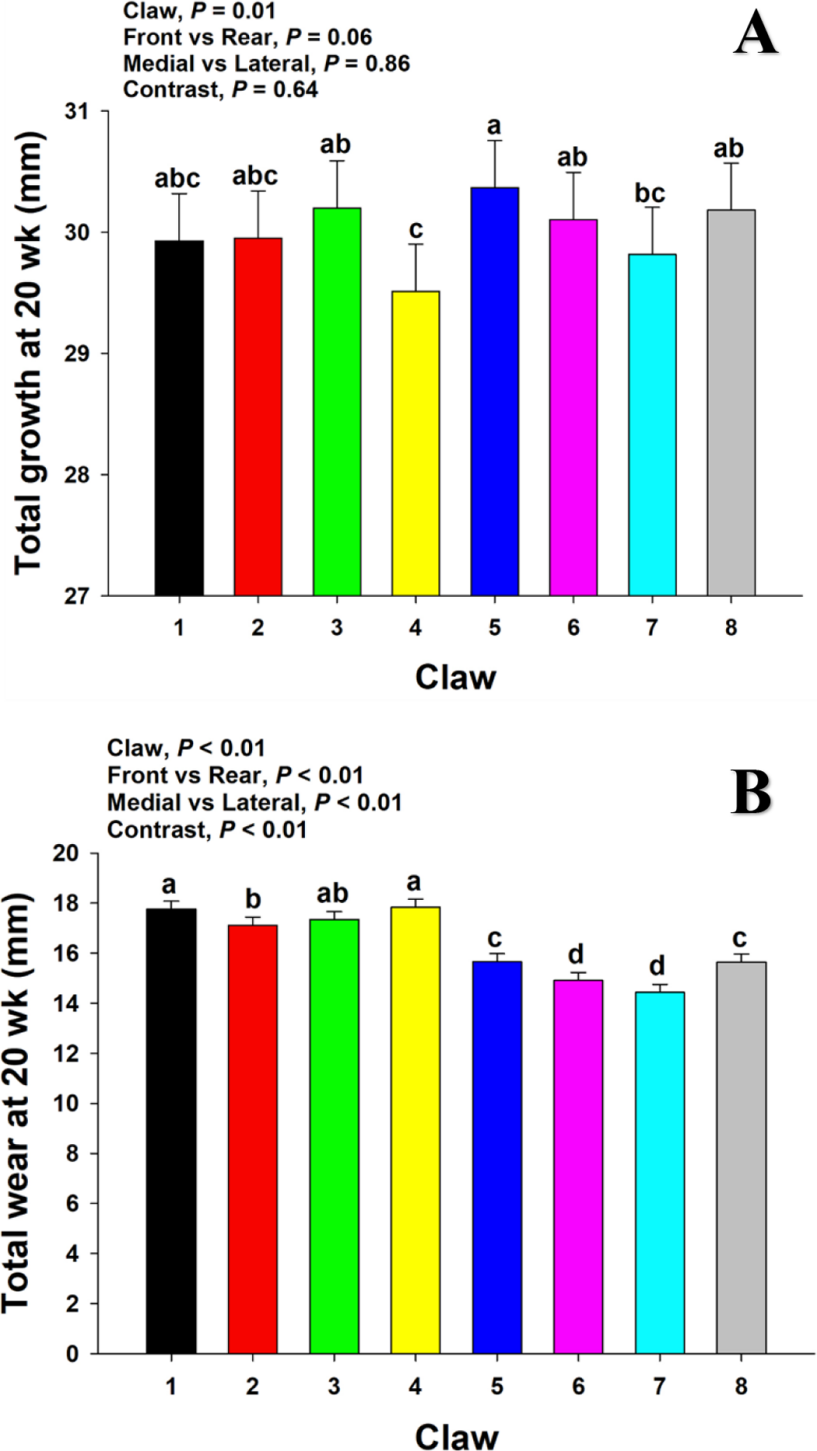
(A) Total hoof growth considered as groove length at 20 wk for each claw. (B) Total wear, calculated by subtracting remnant claw length below the groove line at wk 20 from initial claw length at wk 0. Contrast statements were carried out to compare front versus rear and medial versus lateral claws. The contrast *P*-value denotes the comparison between claws 6 and 7 against all other claws. (a–d) Means without a common lowercase letter are different (*P* < 0.05). Values are means, with SE represented by error bars.

Total wear at wk 20 (Figure 3B) was in direct contrast with claw length at wk 20 (Figure 2B), where greater (*P* < 0.01) wear was observed in front claws than rear (17.5 vs. 15.2 mm) and greater (*P* < 0.01) wear was observed in lateral than medial claws (16.7 vs. 16.0 mm). According to Neveux et al. (2006), mature cows shift most of their weight toward the front legs, and the greater wear in the front legs (Figure 3B) in the current study suggests that perhaps this shift in body weight distribution was already taking place by 20 wk of age. This was also supported by the greater claw length at 20 wk (Figure 2B) in rear claws, which suggests that greater claw length is a product of lower wear in these claws.

Hoof wear is a common concern regarding hoof health, as excessive or variable wear among claw positions may be detrimental to hoof conformation and locomotion. Therefore, hoof wear across several surfaces has been evaluated (Vanegas et al., 2006; Telezhenko et al., 2009). Also, as explained previously, because of differential or inconsistent weight bearing across claws, wear can be substantially reduced in medial front and lateral rear claws (i.e., 2, 3, 5, and 8), which can cause an excessive growth rate with negative effects for such claws if they are not periodically trimmed. Even though the ~1- to 3-mm differences observed throughout this study seem not biologically significant, it is important to understand that these effects are the ultimate visible expression of several underlying interactions and processes at a molecular level, including keratinization. In most cases, keratinization can be negatively affected by various stressors early in life, which persist throughout life and ultimately affect cows' performance, given the vital importance of locomotion needs in the life of dairy cows, such as feed consumption and reproduction.

To the authors' knowledge, this is the first research to describe hoof growth in young dairy cattle. The underlying factors associated with claw length differences at wk 0 remain to be elucidated, but this likely in utero programming may be aimed at providing the necessary ground support for a properly aligned and balanced stance to reach out for food or to be mobile soon after birth. The fact that this uneven claw length effect was maintained 20 wk after birth, even after adjusting for initial claw length as a covariate, strongly underscores the significance of this effect in postnatal hoof growth in dairy calves. The complementary wear to growth explains that there is greater wear in front and lateral claws in young calves, which is translated into lower claw length in the same claws by 20 wk after birth. There is still much to learn on how these claw dynamics (i.e., growth and wear) affect feeding behaviors early in life and the potential repercussions of hoof development in adult animals' lactation performance and reproductive life.
